# Long-term outcomes of ICU-acquired infections with a focus on bloodstream infections: a single-center retrospective registry study

**DOI:** 10.1007/s15010-025-02621-w

**Published:** 2025-08-13

**Authors:** Tero I. Ala-Kokko, Jaana M. Karhu, Pasi Lehto, Sinikka Sälkiö, Pasi Ohtonen, Hannu Syrjälä

**Affiliations:** 1 Research Group of Intensive Care Medicine, Intensive Care Centre, Oulu University Hospital, University of Oulu and Medical Research Center (MRC), PO BOX 29, 90029 Oulu, Finland; 2 The Research Unit of Surgery, Anesthesia and Intensive Care, Oulu University Hospital and University of Oulu, Oulu, Finland; 3 Research Service Unit, Oulu, Finland

**Keywords:** ICU-acquired infections, Long-term outcomes, ICU-acquired bacteremia

## Abstract

**Objectives:**

Intensive care unit (ICU) patients have an increased risk of bacteremia. We aimed to investigate the 5-year outcome of ICU-acquired infections comparing them with ICU patients without new infections. Our second aim was to compare the outcome of Gram-positive, Gram-negative and fungal ICU-acquired bloodstream infections (BSIs).

**Methods:**

This single-center retrospective registry study occurred in an academic teaching hospital during 2000–2017 in a mixed adult ICU consisting of patients who stayed longer than 48 h in the ICU. Data was retrieved from the ICU and hospital electronic data management systems. Three groups were included: no infection and no new antimicrobial treatment, a new ICU-acquired infection with negative blood cultures (BCs), and a new ICU-acquired BSI. A multivariable-adjusted Cox proportional hazards model was used to determine the impact of ICU-acquired infection on 5-year mortality.

**Results:**

1857 had no infection and 768 developed an ICU-acquired infection with positive BCs in 195 cases (25.4%). The adjusted HR was 2.03 (95% CI from 1.76–2.35, p < 0.001) for the impact of ICU-acquired infection on 5-year mortality. The highest median sequential organ failure assessment (SOFA) was 7.0 (5.0–8.0) for the no-infection group, 9.0 (7.0–10.0) for the BC-negative ICU-acquired infection group, and 12.0 (9.0–15.0) for the ICU-acquired BSI patients (p < 0.001). The crude 30-day mortalities in the no-infection, the BC-negative, and the BSI groups were 98 (5.5%), 58 (10.1%), and 51 (26.0%), respectively (p < 0.001). The highest median SOFA for Gram-positive BSIs was 11.0 (8.0–13.0), for Gram-negative BSIs 13.0 (11.0–16.0), and for fungal BSIs 12.5 (10.0–16.0) (p = 0.01). The need for RRT was 23.2% (19) in Gram-positive, 29.8% (14) in Gram-negative, and 48.1% (25) in fungal BSIs (p = 0.01). The crude ICU-mortalities were 12.2% (10) in Gram-positive BSIs, 31.9% (15) in Gram-negative BSIs, and 11.5% (6) in fungal BSIs (p = 0.008). Patients with fungal BSI had the worst 5-year outcome, whereas the long-term outcome did not differ between Gram-positive and Gram-negative BSIs.

**Conclusions:**

Patients with ICU-acquired infections had three times higher 5-year mortality than non-infected ICU patients. ICU-acquired Gram-negative BSIs had the highest ICU mortality, whereas the long-term outcome did not differ between Gram-negative and Gram-positive ICU-acquired BSIs. Fungal BSI showed the worst long-term outcome.

## Introduction

According to a systematic review of population-based studies between 2000 and 2007, the incidence of bloodstream infections (BSI) has been 174–204 per 100,000 person-years in the USA and 166–189 per 100,000 years in Europe [1]. When consecutively collected causes of BSI from 45 nations with more than 200 centers were analyzed between 1997 and 2016, *Escherichia coli* (*E. coli*) and *Staphylococcus aureus* (*S. aureus*) were the predominant organisms of community-onset BSIs worldwide, whereas their order was opposite in hospital-onset BSIs [2].

Among all the patients admitted to ICUs, approximately 5% acquire BSI during ICU stay, as a complication of critical illness or as a consequence of invasive monitoring or using devices for the organ support [3–5]. In a recent study concerning hospital-acquired BSIs 80% were ICU-acquired mainly from respiratory and intravascular catheter infections [6]. It has been found that the ICU-acquired BSIs are independently associated with three- to four-fold increased risk of death [5]. Mortality in patients with ICU-acquired BSIs varies between studies ranging from 36.6–41.1% [5, 7]. The reported attributable mortality for patients with ICU-acquired BSIs has been 2.3–5% according to earlier published papers [5, 7]. In the Prowle et al. study with ICU-acquired BSIs, an independent risk of increased death was associated with *Candida*, *S. aureus* and Gram-negative bacilli BSIs [5].

It has been traditionally considered that Gram-negative BSIs are more severe than Gram-positive BSIs. This idea has been questioned in recent studies, where Gram-positive and Gram-negative BSIs have had almost equal severity, [8] or outcome [9]. In some studies, the short-term outcome has been worse among Gram-negative infections [10]. In general, ICU-acquired BSIs are associated with high morbidity and mortality among critically ill patients, and are found to independently predict mortality [11].

Most previous studies on ICU-acquired BSIs have primarily been focused on short-term outcomes, such as 14-day, 28-day or 30-day mortality, as well as on ICU and hospital mortality rates [5, 12–15] or 1-year mortality [16], whereas the long-term outcomes of the ICU-acquired BSIs are not well documented. Thus, there is a critical gap in the existing literature on the long-term mortality impact of ICU-acquired BSIs. Evaluating long-term mortality is crucial for understanding the full impact of ICU-acquired BSIs on patient outcomes.

The purpose of the present study was to investigate the 5-year outcome of patients with ICU-acquired BSIs compared to patients with blood-culture (BC) negative ICU-acquired infections and those without ICU-acquired infections. We were also interested in whether the hospital and long-term outcome differed between ICU-acquired Gram-positive, Gram-negative and fungal BSIs.

## Methods

### Setting

This registry-based retrospective study was conducted at Oulu University Hospital, which is a 900-bed tertiary-level teaching hospital with 36,000 ward admissions annually. The ICU is a closed medical/surgical unit with a total of 26 beds, five single-bed rooms, two double-bed rooms, and three isolation rooms. The nurse-to-patient ratio varies from 1:1 to 1:2 depending on the severity of patient illnesses and the intensity of treatment. Hand hygiene is based on alcohol hand rub and single-use gloves and gowns are used when in close contact with patients. We have multidisciplinary intensivist-led patient rounds and daily rounds with an infectious disease specialist. Patients are isolated from each other in cases of, for example, suspected or verified influenza, Norovirus, or multidrug resistance (MDR) organisms-associated infections. Protocols are in use to reduce ventilator-associated pneumonia (VAP), catheter-related bloodstream infection (CRBSI), and urinary tract infection (UTI) [17–19].

The study protocol was accepted by the hospital administration (1/2019, 240/2023) and the Digital and Population Data Service Agency (DVV/5100/2023-3).

### Patients

**Fig. 1:**
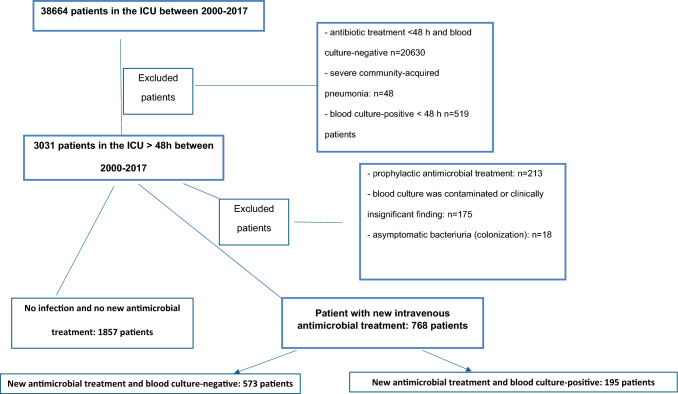
The three groups of patients treated more than 48 h in the ICU

All patients over 18 years who were treated for more than 48 h in our ICU during the years 2000–2017 were included in the study. Patients with prophylactic antimicrobial treatment, contaminated culture result or clinically insignificant findings, antimicrobial treatment on admission or within 48 h, severe community-acquired pneumonia and asymptomatic bacteriuria were excluded (Fig. 1). The diagnosis of infection was based on symptoms, clinical signs, laboratory and microbiological findings, imaging results or surgical findings. The study population was categorized into three groups: no infection and no new antimicrobial treatment, new antimicrobial treatment for a new infection with negative blood cultures, new antimicrobial treatment for a new infection with positive blood cultures. For patients who were readmitted to the ICU, we studied only the first ICU admission during the same hospital stay. The follow-up time for long-term mortality was 5 years.

### Blood cultures

Blood cultures were obtained when a bloodstream infection, bacteremia or fungemia, was clinically suspected. Venous blood (10 ml) was taken into bottles containing neutralizing agents for antimicrobials. Two sets of blood samples were taken at the same time but from different blood vessels. BCs were collected in both aerobic (Bact/Alert FA Plus, Cat# 410851, bioMerieux, Marcy l’Etoile, France) and anaerobic (Bact/Alert FN Plus, Cat# 410852, bioMerieux) blood culture bottles. Bottles were incubated in automated microbial detection system (BactAlert/Virtuo, bioMerieux) for 5 days, and up to 14 days in cases with a suspicion of slowly growing bacteria and yeast. Microbes were identified by standard methods in our certified microbiology laboratory. Positive blood culture bottles were cultured on standard blood, fastidious anaerobe agar (FAA) and chocolate agar plates (Thermo Fisher, Cat#; PO5090A, PB5039A, and PB0225A, respectively). Since 2013, the plate-cultured bacteria were identified with matrix-assisted laser desorption/ionization time-of-flight (MALDI-Tof, Vitek, bioMerieux). *E. coli* and *S. aureus* were identified by chromogenic plate identification. Since the 1990s appropriate breaking points for antibiotic sensitivity have been determined according to the EUCAST-standard by the clinical laboratory [20]. Bacterial and yeast findings were also reported immediately by phone to the clinician. The clinical significance of the finding and contamination was then evaluated considering the clinical picture of the ongoing infection by multidisciplinary team. For common skin contaminants and Gram-positive cocci, at least two positive BCs were required for fulfilling the criterion of BSI. In polymicrobial blood culture findings, the clinically significant causative organism was determined in alignment with the identified infection source.

### Study parameters

The following information was collected for all study patients from the ICU electronic data management system (Centricity Critical Care, GE Healthcare, IL, USA): age, gender, severity of underlying diseases, organ dysfunctions on admission as assessed by the Acute Physiology and Chronic Health Evaluation index (APACHE II) [21]) and organ dysfunction on admission and daily with the total Sequential Organ Failure Assessment (SOFA score) with six different types of organ failure [22], exposure to central venous catheter (CVC) or arterial catheterization before obtaining blood culture; Charlson comorbidity index [23]; surgery before ICU admission; mechanical ventilation (MV); renal replacement therapy (RRT); presence of septic shock [24]; length of ICU and hospital stay, and ICU and hospital mortality. The microbiological data was retrieved from the hospital electronic laboratory patient data record (Weblab Clinical, Nordlab, Finland). The Digital and Population Data Service Agency provided the mortality data regarding those patients who were not alive on 1 May 2023.

### Definitions

ICU-acquired bloodstream infection was defined by an infection onset occurring at least 48 h after ICU admission, with positive blood cultures unrelated to an infection incubating at ICU admission. Antimicrobial treatment was carried out by the treating physicians according to the written hospital antimicrobial guidelines based on updated international literature, local microbial resistance patterns, and daily infectious disease specialist consultation. The antimicrobial treatment was updated according to the results of blood culture findings.

### Statistical analysis

Summary data are presented as means with standard deviations (SDs) or medians with 25th and 75th percentiles. Between-group comparisons for continuous data were performed using analysis of variance (ANOVA) (> 2 groups) and Student’s t-test. Welch’s test was used if the assumption of homoscedastic variances did not hold. Pearson’s χ^2^ test or Fisher’s exact test were used for categorical data. Log-rank (Mantel-Cox) tests were used to compare survival times in univariate analyses. A multivariable adjusted Cox proportional hazards model was used to determine the impact of ICU-acquired infection on 5-year mortality. To minimize the bias in the multivariable model, a directed acyclic graph (DAG) was used to create a minimally sufficient adjustment set. The DAG was drawn using the DAGitty tool [25]. The DAG model indicated that the following parameters should be considered in the Cox model: age, admission year, APACHE II admission score, reason for admission, SOFA score, body mass index, Charlson Comorbidity Index, invasive ventilation, renal replacement therapy and operation before ICU. The results of the Cox model are presented as hazard ratios (HRs) and 95% confidence intervals (CIs). Two-tailed *P* values are reported. Analyses were performed using SPSS for Windows (IBM Corp. Released 2021. IBM SPSS Statistics for Windows, Version 28.0. Armonk, NY: IBM Corp).

## Results

### Patient population

During the study period, 38,664 adult patients were admitted to the ICU of which 3031 stayed longer than 48 h. After exclusion criteria, all together 2625 patients were included in the final analysis: 1875 patients with no infection and 768 patients with ICU-acquired infection (Fig. 1). Blood cultures tested positive in 6.4% (195/3031) among patients with an ICU stay longer than 48 h, which included 25.4% (195/768) of those with ICU-acquired infection. The rate of polymicrobial positive blood cultures was 9.2% (19 out of 195 blood culture-positive cases); 14 with different microbial groups and 5 with the same microbial groups.

#### Comparison of three patient groups whose ICU stay was more than 48 h

There were more females in the no-infection group compared to the infection group (41.8% vs. 30.7%, p < 0.001). The no-infection group also had more often undergone surgery before ICU admission than the ICU-acquired infection group (1395 (75.1%) vs. 459 (59.8%), p < 0.001, Table 1).

**Table 1:** Comparison of three groups who stayed longer than 48 h in a mixed ICU in a university hospital between 2000 and 2017

Parameter	No infection (n = 1857)	Infection with negative blood culture (n = 573)	Infection with positive blood culture (n = 195)	p-value
Sex, female	776 (41.8%)	174 (30.4%)	62 (31.8%)	< 0.001
Age, year	68 (57–74)	67.0 (56.0–74.0)	62.0 (50.0–71.0)	< 0.001
Body mass index	27.0 (24.2–30.4)	26.6 (23.9–30.1)	26.7 (24.2–30.7)	0.78
Charlson comorbidity index	3.0 (2.0–3.0)	3.0 (1.0–3.0)	2.0 (1.0–4.0)	0.65
Malignancy, yes	48 (2.6%)	10 (1.7%)	22 (11.3%)	< 0.001
Admission APACHE II score	17.0 (13.0–22.0)	20.0 (15.0–24.0)	20.0 (16.0–26.0)	< 0.001
Admission SOFA* score	6.0 (4.0–7.0)	7.0 (5.0–8.0)	8.0 (6.0–10.0)	< 0.001
Highest daily SOFA score	7.0 (5.0–8.0)	9.0 (7.0–10.0)	12.0 (9.0–15.0)	< 0.001
Multiorgan failure on ICU admission	490 (27.1%)	238 (42.1%)	107 (55.4%)	< 0.001
Multiorgan failure at any time during the ICU stay	640 (35.1%)	383 (67.3%)	174 (89.2%)	< 0.001
Operation before ICU, yes	1395 (75.1%)	380 (66.3%)	79 (40.5%)	< 0.001
Central venous catheter inserted before blood cultures	5 (0.3%)	10 (1.7%)	184 (94.4%)	< 0.001
Arterial catheter	5 (0.3%)	10 (1.7%)	188 (96.4%)	< 0.001
Mechanical ventilation, yes	1513 (81.5%)	533 (93.0%)	185(94.9%)	< 0.001
Renal replacement therapy, yes	21 (1.1%)	45 (7.9%)	65 (33.3%)	< 0.001
Need of vasopressor	1285 (69.2%)	456 (79.6%)	180 (92.3%)	< 0.001
Site of infection				
Primary blood stream infection	–	N.A.-	16 (8.2%)	
Clinical sepsis	–	16 (2.8%)	N.A.	
Catheter-related blood stream infection	–	N.A.	20 (10.3%)	
Respiratory	–	514 (89.7%)	97 (49.7%)	
Intraabdominal	–	10 (1.7%)	32 (16.4%)	
Skin and soft tissue	–	6 (1.0%)	17 (8.7%)	
Urinary tract infection	–	20 (3.5%)	0 (0%)	
Central nervous system infection	–	5 (0.9%)	4 (2.1%)	
Other^a^	–	2 (0.3%)	9 (4.6%)	
ICU stay, days	2.9 (2.4–3.8)	5.9 (4.2–9.2)	12.9 (7.6–20.4)	< 0.001
Hospital stay, days	9.0 (7.0–13.0)	13.0 (9.0–20.0)	27.0 (19.0–42.0)	< 0.001
ICU mortality	38 (2.0%)	18 (3.1%)	33 (16.9%)	< 0.001
30-day mortality	98 (5.3%)	58 (10.1%)	51 (26.2%)	< 0.001

The no-infection group did not receive new antimicrobials, whereas the ICU-acquired infection group received new antimicrobials, while their blood cultures were negative or positive. Numbers are either per cents or medians with 25 and 75 percentiles or means and standard deviation (SD)

*N.A.* not applicable

^a^Includes 2 mediastinitis, 3 wound infections, 2 endocarditis and unknown 4

Those with ICU-acquired infection were more severely ill on admission than the no-infection group, had more severe organ dysfunctions during the ICU stay and needed more of the following organ support therapies: mechanical ventilation (718 (93.5%) vs. 1513 (81.5%), p < 0.001), vasoactive support (636 (82.8%) vs.1285 (69.2%), p < 0.001) and RRT (110 (14.3%) vs. 21 (1.1%), p < 0.001). ICU mortality was higher in the ICU-acquired infection group compared to the no-infection group (6.6% vs. 2.0%, p < 0.001, Table 1).

When BC-positive and BC-negative groups were compared, the patients with BC-negative ICU-acquired infections were older than patients with BSIs (67.0 (56.0–74.0) vs. 62.0 (50.0–71.0), p < 0.001). They also had more often undergone operations before ICU admission than ICU-acquired BSI patients (380 (66.3%) vs. 79 (40.5%), p < 0.001, Table 1). The following variables did not differ between BC-positive and BC-negative groups: Admission APACHE II score (20.0 (15.0–24.0) vs. 20.0 (16.0–26.0, p = 0.10); the need for mechanical ventilation (533 (93.0%) vs. 185 (94.9%), p = 0.41).

The patients with BC-positive infections had the most severe organ dysfunctions on admission (Table 1). SOFA was 6.0 (4.0–8.0) for the no-infection group, 7.0 (5.0–8.0) for BC-negative ICU-acquired infections and 8.0 (6.0–10.0) for ICU-acquired BSI patients (p < 0.001). During the ICU stay SOFA was 7.0 (6.0–8.0) for the no-infection group, 9.0 (7.0–10.0) for BC-negative ICU-acquired infections and 12.0 (9.0–15.0) for ICU-acquired BSI patients (p < 0.001, Table 1). BSI patients most often required renal replacement therapy (21 (1.1%) for the no-infection group, 45 (7.9%) for BC-negative ICU-acquired infections, and 65 (33.3%) for BSI patients, p < 0.001). Also, BSI patients more often had central venous catheters than the patients with BC-negative infection (94.4% vs. 1.7%, p < 0.001). ICU-acquired BSI patients had more malignancies than patients with BC-negative infections (11.3% vs.1.7%, p < 0.001). When infection sites were compared (Table 1), BSI patients had fewer respiratory infections than BC-negative patients (89.7% vs. 49.7%). They had more intra-abdominal infections (16.4% vs.1.7%) and skin and soft tissue infections (8.7% vs.1.0%) than BC-negative ICU-acquired infection patients.

#### Five-year survival analysis of the three study groups

**Fig. 2:**
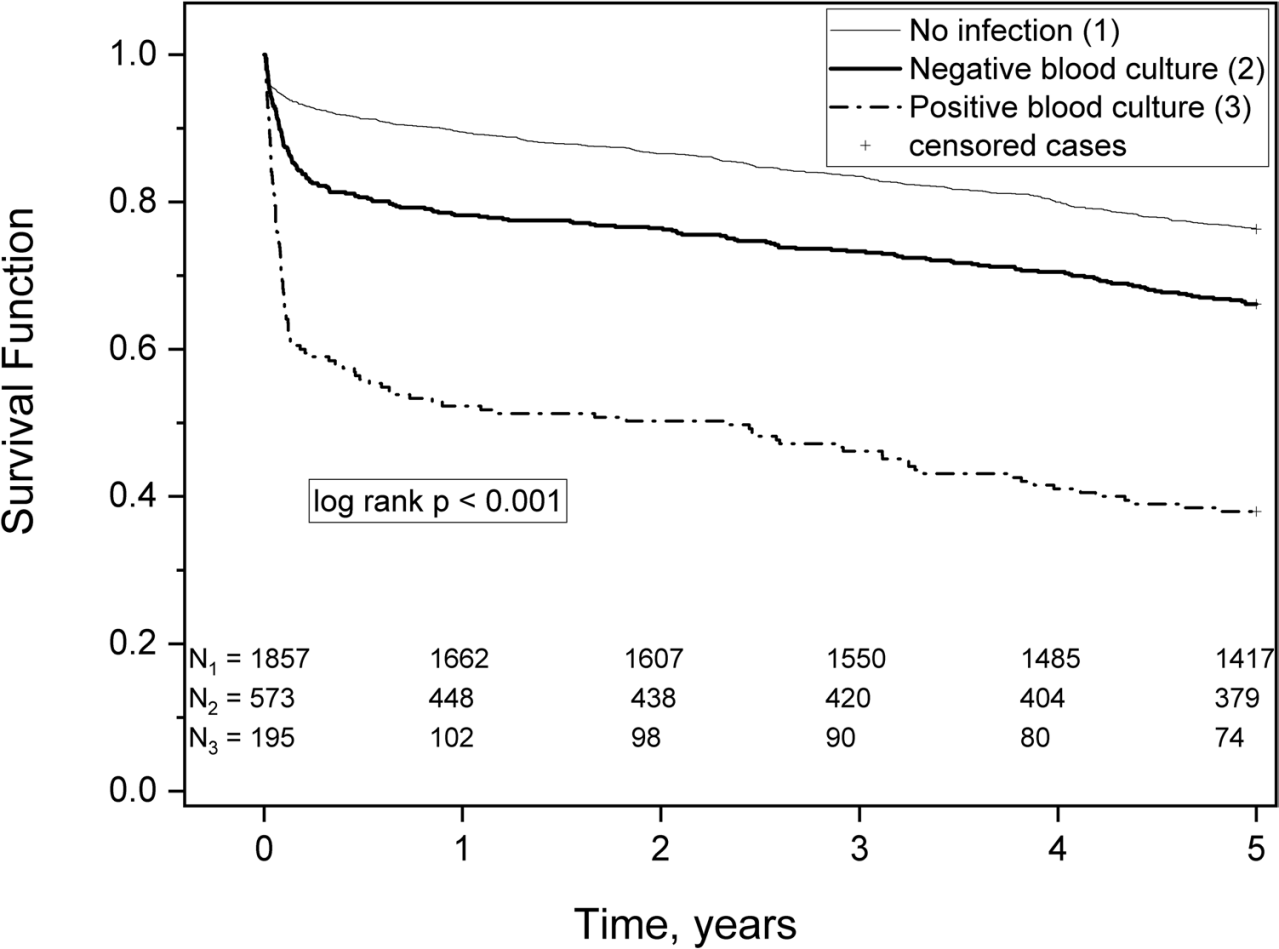
Comparison of survival of three groups: no infection, new antimicrobial treatment and blood culture-negative infection (573 patients) and new antimicrobial treatment and blood culture-positive infection (195 patients)

**Fig. 3:**
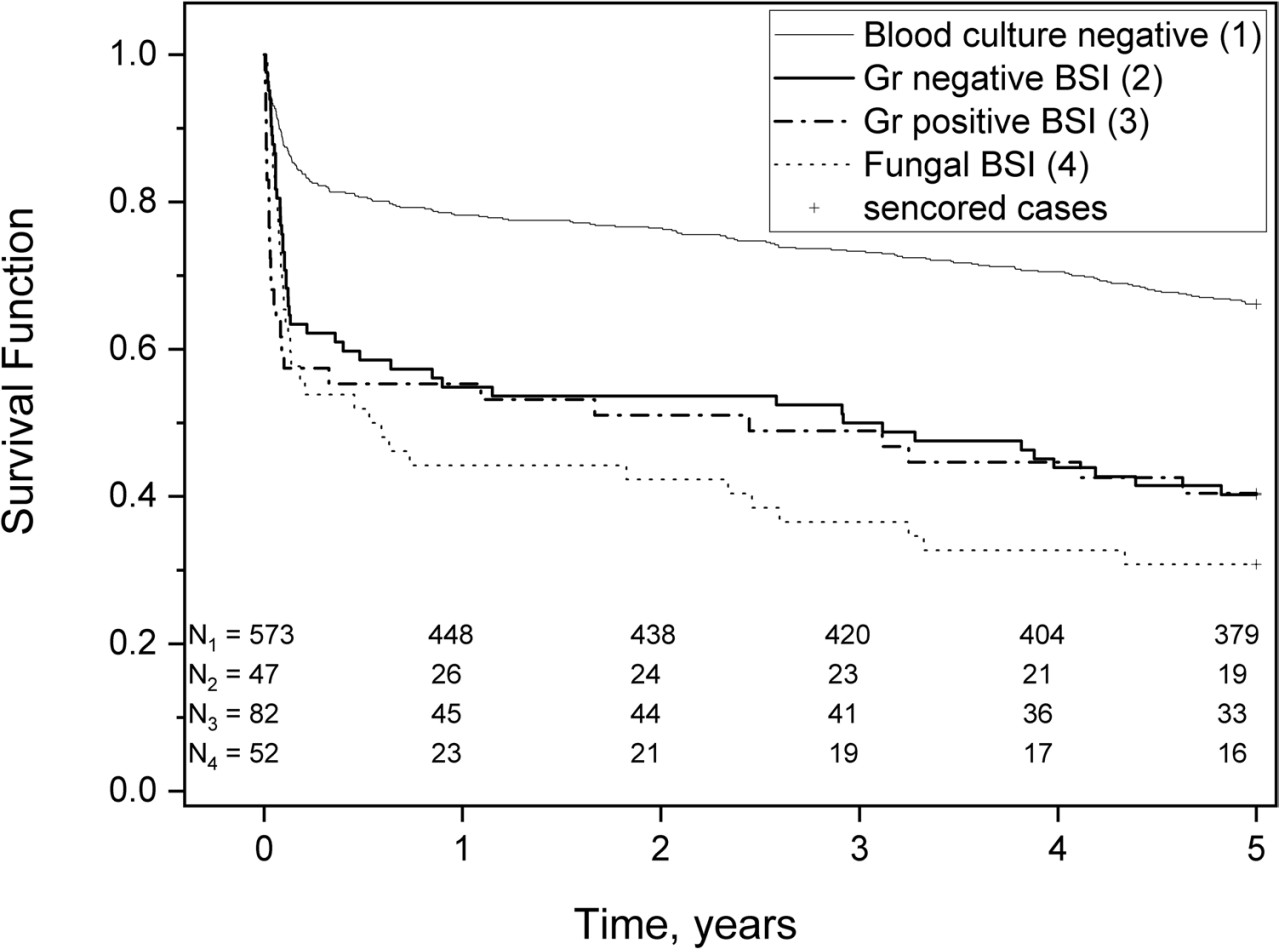
Comparison of 5-year survival within four groups: blood culture-negative infection, Gram-negative blood stream infection (BSI), Gram-positive BSI, and fungal BSI

As the Kaplan–Meier survival analysis in Fig. 2 shows, there were statistically significant differences in survival between the three study groups (p < 0.001). Long-term survival was poorest among ICU-acquired BSI patients, less severe among patients with BC-negative infections and most favorable in the no-infection group. Mortality was highest within two months after ICU-admission. When infection patients’ survival curves were compared (Fig. 3), patients with ICU-acquired BSI had statistically significant survival curve drops compared to patients with BC-negative infections (p < 0.001).

When 5-year mortality was compared using the no-infection group as a reference, the unadjusted HR of the Cox proportional hazards model for the impact of ICU-acquired infection on 5-year mortality was 1.37 (95% CI 1.15–1.63, p < 0.001). According to the adjusted Cox proportional hazards model, the HR was 2.03 (95% CI from 1.76–2.35, p < 0.001).

#### Comparison of blood culture-positive ICU-acquired infections

As Table 2 shows, altogether 195 microorganisms were isolated from ICU-acquired BSIs. Gram-positive cocci were the most common group (N = 82, 42.1%). The most often isolated bacteria were *S. aureus* (n = 30, 15.4%), and *Enterococcus faecium* (N = 22, 11.3%). Yeasts were the second most common group (n = 52, 26.7%) with *Candida albicans* the most frequently occurring species (n = 40, 20.5%). There were 47 Gram-negative bacilli (24.1%), of which *Enterobacter cloacae* (n = 13, 6.7%), *Pseudomonas aeruginosa* (n = 7, 3.6%), and *Serratia marcescens* (n = 7, 3.6%) were identified most often. The number of other microorganisms in other microbe groups totaled less than 10 (Table 2).

**Table 2:** 195 microorganisms isolated from the blood cultures in ICU-acquired infections between 2000 and 2017 in a mixed ICU in a university hospital

Microbe groups	Number	Per cent
Gram-positive cocci	82	42.1
*Enterococcus faecalis*	5	2.6
*Enterococcus faecium*	22	11.3
*Enterococcus gallinarum*	1	0.5
*Leuconostoc lactis*	1	0.5
*Staphylococcus aureus*^a^	30	15.4
*Staphylococcus capitis*	1	0.5
*Staphylococcus epidermidis*	18	9.2
*Staphylococcus lugdunensis*	2	1.0
*Staphylococcus hominis*	1	0.5
*Streptococcus anginosus*	1	0.5
Gram-negative bacilli	47	24.1
*Acinetobacter baumannii*	2	1.0
*Chryseobacterium meningosepticum*	1	0.5
*Escherichia coli*	6	3.1
*Enterobacter cloacae*	13	6.7
*Klebsiella oxytoca*	2	1.0
*Klebsiella pneumoniae*	4	2.1
*Klebsiella terrigena*	1	0.5
*Morganella morganii*	1	0.5
*Pseudomonas aeruginosa*	7	3.6
*Serratia marcescens*	7	3.6
*Serratia rubidaea*	1	0.5
*Stenotrophomonas maltophilia*	2	1.0
Gram-positive bacilli	5	2.6
*Bacillus cereus*	4	2.0
*Microbacterium* spp.	1	0.5
Anaerobes	9	4.6
*Bacteroides fragilis*	1	0.5
*Bacteroides fragilis group*	2	1.0
*Bacteroides thetaiotaomicron*	3	1.5
*Eubacterium lentum*	2	1.0
*Lactobacillus* spp.	1	0.5
Yeast	52	26.7
*Candida albicans*	40	20.5
*Candida glabrata*	5	2.6
*Candida inconspicua*	1	0.5
*Candida krusei*	2	1.0
*Candida parapsilosis*	2	1.0
*Candida tropicalis*	1	0.5
*Saccharomyces cerevisiae*	1	0.5

^a^All were methicillin-susceptible *Staphylococcus aureus* (MSSA-type)

Table 3 shows the comparisons between the abovementioned three most common microbe groups. The patients in the Gram-negative BSI group had the highest admission SOFA score (p = 0.05) and the highest daily SOFA score (p = 0.01). These patients had more often multiorgan failure on admission (p = 0.07) and had undergone surgical procedure before ICU admission (p = 0.048). Patients with fungal sepsis needed more often RRT (48.1%, p = 0.01).

**Table 3:** Comparison of three blood culture-positive groups who had an ICU-acquired infection and who stayed longer than 48 h in a mixed ICU in a university hospital between 2000 and 2017

Parameter	Gram-positive cocci (n = 82)	Gram-negative bacilli (n = 47)	Yeast (n = 52)	p-value
Sex, female	26 (31.7%)	10 (21.3%)	21 (40.4%)	0.12
Age, year	64.1 (52.4–72.0)	62.0 (48.4–72.9)	58.8 (46.5–69.0)	0.52
Body mass index	26.8 (24.6–31.1)	26.8 (22.3–29.3)	26.6 (24.4–31.1)	0.69
Charlson comorbidity index	2.0 (1.0–3.0)	2.0 (1.0–4.0)	3.0 (1.0–4.0)	0.92
Malignancy, yes	12 (14.6%)	4 (8.5%)	6 (11.5%)	0.59
Admission APACHE II score	20.0 (16.0–26.0)	19.0 (16.0–26.5)	20.0 (15.5–25.0)	0.95
Admission SOFA score	7.0 (5.0–9.0)	9.0 (6.0–10.0)	8.0 (6.0–11.0)	0.05
Highest daily SOFA score	11.0 (8.0–13.0)	13.0 (11.0–16.0)	12.5 (10.0–16.0)	0.01
Multiorgan failure on admission	40 (48.8%)	29 (63.0%)	29 (56.9%)	0.07
Multiorgan failure at any time during the ICU stay	68 (82.9%)	45 (95.7%)	48 (92.3%)	0.75
Operation before ICU, yes	31 (37.8%)	23 (48.9%)	20 (38.5%)	0.048
Central venous catheter insertion before blood cultures	75 (915%)	45 (95.7%)	50 (96.2%)	0.56
Arterial catheter	80 (97.6%)	46 (97.9%)	48 (92.3%)	0.30
Mechanical ventilation, yes	79 (96.3%)	44 (93.6%)	48 (92.3%)	0.64
Renal replacement therapy, yes	19 (23.2%)	14 (29.8%)	25 (48.1%)	0.01
Need of vasopressor	73 (89.0%)	46 (97.9%)	48 (92.3%)	0.22
Site of infection				0.56
Catheter-related blood stream infection	8 (9.8%)	3 (6.4%)	9 (17.3%)	
Blood stream	6 (7.3%)	4 (8.5%)	5 (9.6%)	
Respiratory	44 (53.7%)	25 (53.2%)	22 (42.3%)	
Intraabdominal	9 (11.0%)	10 (21.3%)	7 (13.5%)	
Skin and soft tissue	7 (8.5%)	4 (8.5%)	5 (9.6%)	
Central nervous system infections	2 (2.4%)	0	2 (4.8%)	
Other	6 (7.3%)	1 (2.1%)	2 (3.8%)	
ICU stay, days	11.3 (7.0–19.0)	11.8 (6.7–15.1)	16.1 (10.6–21.4)	0.42
Hospital stay, days	27 (19.0–39.5)	19 (13.0–24.0)	32.5(26.0–65.0)	0.022
ICU mortality	10 (12.2%)	15 (31.9%)	6 (11.5%)	0.008
30-day mortality	16 (19.5%)	17 (36.2%)	14 (26.9%)	0.12

Numbers are either per cent or medians with 25 and 75 percentiles or means and standard deviation (SD)

The fungal BSI group had most often catheter related BSIs (17.3%), while intra-abdominal site was the most often seen in Gram-negative BSIs (21.3%). Respiratory origin was the most common site of BSI in all three groups: Gram-positive BSIs; 53.7%, Gram-negative BSIs; 53.2% and fungal BSIs; 42.3%.

The ICU mortality was highest among patients with Gram-negative BSIs (31.9%), while the corresponding figure was 12.2% for the patients with Gram-positive BSI and for the yeast group 11.5% (p = 0.008) (Table 3.). The mean hospital stay for fungal patients was 43.8 days, for Gram-negative BSIs 24.6 days, and for the Gram-positive cocci 31.1 days (p = 0.022). The crude 30-day mortality was still highest, but no longer statistically significant, in Gram-negative BSIs (17 out of 47 cases, 36.2%). In fungal BSIs crude 30-day mortality was 14 out of 52 cases (26.9%) and in Gram-positive BSIs 16 (19.5%) out of 82 cases (p = 0.12).

The Kaplan–Meier survival analysis shows early mortality curve separation within two months from ICU admission. There was no difference in the long-term survival between Gram-positive and Gram-negative BSIs. Patients with fungal BSIs had the worst 5-year survival. The survival curves of Gram-negative and Gram-positive BSIs separated during the first months. Thereafter, the curves mimicked each other, while fungal BSIs curves continued to decrease during the first year (Fig. 3). After that, the curves in these groups had similar slopes.

## Discussion

The results of the present study suggest that ICU-acquired BSIs are associated with an increased long-term risk of death compared to those without infection or BC-negative ICU-acquired infection. Gram-negative ICU-acquired BSIs were associated with higher ICU mortality compared to Gram-positive BSIs and fungal BSIs.

Previous short-term studies often focus on specific time points, such as 28-day mortality. However, many survivors at these points remain in the ICU or are discharged but not yet fully recovered [14]. Complications, recurrent infections, or underlying comorbidities can influence post-discharge outcomes. To our knowledge, this is the first study to investigate the 5-year outcomes of ICU-acquired BSIs, comparing results of BC-negative ICU-acquired infections and patients without ICU-acquired infections. Previous studies on severe sepsis found most mortality occurs in the hospital, and within the first year with a continued decline over 5 years [26]. However, these studies did not specifically investigate ICU-acquired BSIs.

In our series, ICU-acquired BSI patients had more than five-fold higher ICU mortality compared to BC-negative ICU-acquired infections. This difference continued up to 5 years. For example, 89.2% of BSI patients had multiple organ failure during ICU stay, and 11.3% had malignancy compared to only 1.7% of BC-negative patients. Although 94% of patients with BC- positive infection had an exposure to CVC catheters before obtaining blood cultures only 10% had catheter related infection. Our figure is lower than published earlier in literature [27]. In our unit we have used several years a prevention protocol for prevention of CVC infections [18]. Conflicting results have been reported regarding the influence of factors such as infection source, co-morbidities, illness severity, pathogens, and their susceptibility profiles on ICU-BSI mortality. For example, Adrie and colleagues found ICU-acquired BSI increased the risk of death by 40%, especially with Gram-negative infections, pneumonia, or unknown infection sites, and inadequate initial treatment [28]. The fungal infections results had the worst 30-day mortality, which continued up to 5 years and is similar to our series. Others have found that Gram-negative BSIs are linked to more severe clinical conditions, with higher incidences of septic shock and severe sepsis compared to Gram-positive BSIs, but without significantly different mortality rates between Gram-negative and Gram-positive infections [29]. In our series, Gram-negative bacteremia caused more severe organ dysfunctions, but long-term outcomes did not differ between Gram-negative and Gram-positive groups.

Ongoing antimicrobial treatment is one of the confounding factors when assessing the effect of bacteremia on mortality [30]. In our series, blood cultures were drawn before the initiation of new antimicrobial treatment. In our series, 92% of bacteremia patients and 80% of BC-negative patients had septic shock. Those with bacteremia had significantly more severe organ dysfunction, with a higher proportion requiring renal replacement therapy. The 30-day mortality of bacteremic patients was higher compared to BC-negative patients (26% vs. 10%).

The foci of infections influence disease progression and clinical outcomes. For example, lung, skin and soft tissue infections and peritonitis have been considered to have a worse prognosis [31]. In our series, respiratory infections were the most common focus in both BSI and BC-negative patients. On the other hand, none of our BC-positive patients had urinary tract infections.

In our series with ICU-acquired bacteremia, there was no difference in long-term survival between Gram-positive and Gram-negative bacteremias. Others have shown in both community- and ICU-acquired BSI that higher serum inflammatory factor concentrations and greater disease severity are associated with Gram-negative sepsis compared to sepsis caused by Gram-positive bacteria [9, 29]. In our series of ICU-acquired BSI there was a predominance of Gram-positive microbes, with *S. aureus* being the most frequent bacteria. An augmented host inflammatory response has been found in *S. aureus* infections compared with *E. coli* in an ex vivo model [32]. On the contrary, in intra-abdominal sepsis the cytokine response is clearly higher in Gram-negative compared to in Gram-positive bacteremia [33]. Our patients with ICU-acquired Gram-negative BSI had higher ICU mortality (31.9%) compared to Gram-positive BSI cases (12.2%), but this difference disappeared in the long term.

Recent studies have had conflicting results regarding the effect of Gram-positive and Gram-negative bacteremia on mortality [9, 10]. According to the retrospective ICU sepsis data, Gram-negative bacteria and fungi were independent risk factors for poorer 28-day survival rate in multivariable analysis [10]. However, as the authors commented, patients with positive etiological tests might not accurately represent the cause of their infection, because colonization bacteria and opportunistic pathogens may have interfered with the results. In the other meta-analysis concerning sepsis studies published between 2003 and 2023, sepsis survival rate did not differ between Gram-positive or Gram-negative bacteria [9]. Our series found that Gram-negative bacteremia caused more severe organ dysfunctions, explaining the higher short-term mortality.

It has been shown that BC-positive sepsis patients requiring RRT carries a high mortality risk of 50% [34]. In our series, the need for RRT in Gram-positive BSI was 23%, in Gram-negative BSI 30% and in fungal BSI 48%, which had the worst long-term outcome. The poor outcome with fungal BSIs is in the line with an earlier study, where increasing mortality continued during a 4-year follow-up period [35]. Interestingly, renal failure was a good predictor of 4-year mortality in these patients [35].

Taken together, earlier literature and our results suggest that differences in host responses, immune balance, need of RRT, foci of infections and the spectrum of microorganisms may explain the difference in outcomes of ICU-acquired BSIs.

### Limitations and strengths

One of the primary limitations of this study was its single-center retrospective cohort design, which carries inherent drawbacks. Our data was originally not gathered for this study, but is based on existing registry data. The findings may not be entirely representative of current clinical practices due to the 17-year study period. However, the admission year was included in the multivariate model as an adjusting factor. This, together with the fact that the data were originally gathered for clinical purposes, means there is a risk for selection and confounding bias as well as insufficient coding of infections. The relatively small number of BSIs in different microbe groups limits the study’s conclusions.

However, the main strength is our relatively large patient population with a uniform electronic patient data system in a single center. All blood culture findings were rechecked by an infectious disease physician to exclude colonization. Opportunistic microbes were included only if present in at least two different blood cultures. Although this was a register study, our daily practice during the study period included a multidisciplinary team with intensivists, an infectious diseases physician and radiologist. The team planned the diagnostic investigations and made patient treatment decisions.

### Clinical impact and need of future studies

To our knowledge, this is one of the few studies focusing specifically on the long-term mortality following hospital-acquired infections in ICU settings while the focus of many earlier studies has been on short-term outcomes (e.g., ICU-and hospital mortality, 28-day mortality). Given the increasing ICU survival rates due to advances in critical care, understanding the long-term consequences of ICU-acquired BSIs is essential. The markedly higher mortality associated with gram-negative and fungal ICU acquired BSIs and significantly increased length of hospital stay underscore the importance of following prevention protocols in clinical practice. Thus far in the literature the prevention strategies include stringent infection control practices, timely removal of unnecessary catheters, and appropriate antimicrobial therapy [14, 36–38]. Future research should also focus on elucidating the immunological and microbiological mechanisms underlying the observed long-term mortality differences between Gram-positive, Gram-negative and fungal BC-positive infections. Furthermore, our findings underscore the need for extended follow-up, highlighting the importance of assessing long-term consequences (trajectories) beyond short-term mortality.

In conclusion, among patients who stayed more than 48 h in the ICU, those with ICU-acquired infections had three times higher 5-year mortality than non-infected ICU patients. ICU mortality was higher among Gram-negative BSIs compared to Gram-positive BSIs and fungal BSIs. The survival difference between Gram-negative and Gram-positive BSIs disappeared within two months, while fungal BSIs showed decreased survival up to 1 year and remained lower 5 years later.

## Data Availability

All data generated or analyzed during this study are included in this published article.

## Abbreviations

ICU: Intensive care unit
BSI: Bloodstream infection
BC: Blood culture
CVC: Central venous catheter
HR: Hazard ratio
SOFA: Sequential organ failure assessment
RRT: Renal replacement therapy
MDR: Multidrug-resistant
MSSA: Methicillin-susceptible *Staphylococcus aureus*
VAP: Ventilator-associated pneumonia
CRBSI: Catheter-related bloodstream infection
UTI: Urinary tract infection
FAA: Fastidious anaerobe agar
APACHE II: Acute Physiology and Chronic Health Evaluation II
CVC: Central venous catheter
MV: Mechanical ventilation
DAG: Directed acyclic graph
CI: Confidence interval
SD: Standard deviation

## References

[1] Goto M, Al-Hasan MN. Overall burden of bloodstream infection and nosocomial bloodstream infection in North America and Europe. Clin Microbiol Infect. 2013;19(6):501–9. 10.1111/1469-0691.12195. 23473333 10.1111/1469-0691.12195

[2] Diekema DJ, Hsueh PR, Mendes RE, Pfaller MA, Rolston KV, Sader HS, Jones RN. The microbiology of bloodstream infection: 20-year trends from the SENTRY antimicrobial surveillance program. Antimicrob Agents Chemother. 2019;63(7):e00355-e419. 10.1128/AAC.00355-19. 31010862 10.1128/AAC.00355-19 PMC6591610

[3] Timsit JF, Laupland KB. Update on bloodstream infections in ICUs. Curr Opin Crit Care. 2012;18(5):479–86. 10.1097/MCC.0b013e328356cefe. 22820156 10.1097/MCC.0b013e328356cefe

[4] Vincent JL, Rello J, Marshall J, Silva E, Anzueto A, Martin CD, Moreno R, Lipman J, Gomersall C, Sakr Y, Reinhart K, EPIC II Group of Investigators. International study of the prevalence and outcomes of infection in intensive care units. JAMA. 2009;302(21):2323–9. 10.1001/jama.2009.1754. 19952319 10.1001/jama.2009.1754

[5] Prowle JR, Echeverri JE, Ligabo EV, Sherry N, Taori GC, Crozier TM, Hart GK, Korman TM, Mayall BC, Johnson PD, Bellomo R. Acquired bloodstream infection in the intensive care unit: incidence and attributable mortality. Crit Care. 2011;15(2):R100. 10.1186/cc10114. 21418635 10.1186/cc10114 PMC3219371

[6] Buetti N, Tabah A, Setti N, Ruckly S, Barbier F, Akova M, Tarik Aslan A, Leone M, Bassetti M, Conway Morris A, Arvaniti K, Paiva J-A, Ferrer R, Qiu H, Montrucchio G, Cortegiani A, Kayaaslan B, De Bus L, De Waele JJ, Jean-François Timsit J-F, EUROBACT-2 Study Group, the European Society of Intensive Care Medicine (ESICM), the European Society of Clinical Microbiology, the Infectious Diseases (ESCMID) Study Group for Infections in Critically Ill Patients (ESGCIP), and the OUTCOMEREA Network. The role of centre and country factors on process and outcome indicators in critically ill patients with hospital-acquired bloodstream infections. Intensive Care Med. 2024;50(6):873–89. 10.1007/s00134-024-07348-0. 38498170 10.1007/s00134-024-07348-0 PMC11164726

[7] Massart M, Wattecamps G, Moriconi M, Fillatre P. Attributable mortality of ICU-acquired bloodstream infections: a propensity-score matched analysis. Eur J Clin Microbiol Infect Dis. 2021;40:1673–80. 10.1007/s10096-021-04215-4. 33694037 10.1007/s10096-021-04215-4 PMC7945601

[8] Verway M, Brown KA, Marchand-Austin A, Diong C, Lee S, Langford B, Schwartz KL, MacFadden DR, Patel SN, Sander B, Johnstone J, Garber G, Daneman N. Prevalence and mortality associated with bloodstream organisms: a population-wide retrospective cohort study. J Clin Microbiol. 2022;60(4):e0242921. 10.1128/jcm.02429-21. 35254101 10.1128/jcm.02429-21 PMC9020345

[9] Tang A, Shi Y, Dong Q, Wang S, Ge Y, Wang C, Gong Z, Zhang W, Chen W. Prognostic differences in sepsis caused by gram-negative bacteria and gram-positive bacteria: a systematic review and meta-analysis. Crit Care. 2023;27(1): 467. 10.1186/s13054-023-04750-w. 38037118 10.1186/s13054-023-04750-w PMC10691150

[10] Guo Q, Qu P, Cui W, Liu M, Zhu H, Chen W, Sun N, Geng S, Song W, Li X, Lou A. Organism type of infection is associated with prognosis in sepsis: an analysis from the MIMIC-IV database. BMC Infect Dis. 2023;23(1): 431. 10.1186/s12879-023-08387-6. 37365506 10.1186/s12879-023-08387-6 PMC10291766

[11] Laupland KB, Kirkpatrick AW, Church DL, Ross T, Gregson DB. Intensive-care-unit-acquired bloodstream infections in a regional critically ill population. J Hosp Infect. 2004;58(2):137–45. 10.1016/j.jhin.2004.04.031. 15474185 10.1016/j.jhin.2004.06.007

[12] Wang YC, Shih SM, Chen YT, Hsiung CA, Kuo SC. Clinical and economic impact of intensive care unit-acquired bloodstream infections in Taiwan: a nationwide population-based retrospective cohort study. BMJ Open. 2020;10(11): e037484. 10.1136/bmjopen-2020-037484. 33243790 10.1136/bmjopen-2020-037484 PMC7692834

[13] Kallel H, Houcke S, Resiere D, Roy M, Mayence C, Mathien C, Mootien J, Demar M, Hommel D, Djossou F. Epidemiology and prognosis of intensive care unit-acquired bloodstream infection. Am J Trop Med Hyg. 2020;103(1):508–14. 10.4269/ajtmh.19-0877. 32314689 10.4269/ajtmh.19-0877 PMC7356483

[14] Tabah A, Buetti N, Staiquly Q, et al. Epidemiology and outcomes of hospital-acquired bloodstream infections in intensive care unit patients: the EUROBACT-2 international cohort study. Intensive Care Med. 2023;49:178–90. 10.1007/s00134-022-06944-2. 36764959 10.1007/s00134-022-06944-2 PMC9916499

[15] Lim SJ, Choi JY, Lee SJ, Cho YJ, Jeong YY, Kim HC, Lee JD, Hwang YS. Intensive care unit-acquired bloodstream infections: a 5-year retrospective analysis of a single tertiary care hospital in Korea. Infection. 2014;42(5):875–81. 10.1007/s15010-014-0651-z. 25030309 10.1007/s15010-014-0651-z

[16] McNamara JF, Righi E, Wright H, Hartel GF, Harris PNA, Paterson DL. Long-term morbidity and mortality following bloodstream infection: a systematic literature review. J Infect. 2018;77(1):1–8. 10.1016/j.jinf.2018.04.005. 29746948 10.1016/j.jinf.2018.03.005

[17] Jansson M, Ala-Kokko T, Ahvenjärvi L, Karhu J, Ohtonen P, Syrjälä H. What is the applicability of a novel surveillance concept of ventilator-associated events? Infect Control Hosp Epidemiol. 2017;38(8):983–8. 10.1017/ice.2017.106. 28612697 10.1017/ice.2017.106

[18] O’Grady NP, Alexander M, Burns LA, Dellinger EP, Garland J, Heard SO, Lipsett PA, Masur H, Mermel LA, Pearson ML, Raad II, Randolph AG, Rupp ME, Saint S, Healthcare Infection Control Practices Advisory Committee. Guidelines for the prevention of intravascular catheter-related infections. Am J Infect Control. 2011;39(4 Suppl 1):S1-34. 10.1016/j.ajic.2011.01.003. 21511081 10.1016/j.ajic.2011.01.003

[19] Tyson AF, Campbell EF, Spangler LR, Ross SW, Reinke CE, Passaretti CL, Sing RF. Implementation of a nurse-driven protocol for catheter removal to decrease catheter-associated urinary tract infection rate in a surgical trauma ICU. J Intensive Care Med. 2020;35(8):738–44. 10.1177/0885066618781304. 29886788 10.1177/0885066618781304

[20] The European Committee on Antimicrobial Susceptibility Testing. Breakpoint tables for interpretation of MICs and zone diameters. Version 15.0. 2025. https://www.eucast.org.

[21] Knaus WA, Draper EA, Wagner DP, Zimmerman JE. APACHE II: a severity of disease classification system. Crit Care Med. 1985;13(10):818–29. 3928249

[22] Vincent JL, de Mendonça A, Cantraine F, Moreno R, Takala J, Suter PM, Sprung CL, Colardyn F, Blecher S. Use of the SOFA score to assess the incidence of organ dysfunction/failure in intensive care units: results of a multicenter, prospective study. Crit Care Med. 1998;26(11):1793–800. 10.1097/00003246-199811000-00016. 9824069 10.1097/00003246-199811000-00016

[23] Charlson ME, Pompei P, Ales KL, MacKenzie CR. A new method of classifying prognostic comorbidity in longitudinal studies: development and validation. J Chron Dis. 1987;40(5):373–83. 10.1016/0021-9681(87)90171-8. 3558716 10.1016/0021-9681(87)90171-8

[24] Levy MM, Fink MP, Marshall JC, Abraham E, Angus D, Cook D, Cohen J, Opal SM, Vincent JL, Ramsay G, SCCM/ESICM/ACCP/ATS/SIS. 2001 SCCM/ESICM/ACCP/ATS/SIS international sepsis definitions conference. Crit Care Med. 2003;31(4):1250–6. 10.1097/01.CCM.0000050454.01978.3B. 12682500 10.1097/01.CCM.0000050454.01978.3B

[25] Textor J, van der Zander B, Gilthorpe MS, Liskiewicz M, Ellison GT. Robust causal inference using directed acyclic graphs: the R package ‘dagitty.’ Int J Epidemiol. 2016;45(6):1887–94. 10.1093/ije/dyw341. 28089956 10.1093/ije/dyw341

[26] Cuthbertson BH, Elders A, Hall S, Taylor J, MacLennan G, Mackirdy F, Mackenzie SJ, Scottish Critical Care Trials Group, Scottish Intensive Care Society Audit Group. Mortality and quality of life in the five years after severe sepsis. Crit Care. 2013;17(2): R70. 10.1186/cc12616. 23587132 10.1186/cc12616 PMC4057306

[27] Stewart AG, Laupland KB, Tabah A. Central line associated and primary bloodstream infections. Curr Opin Crit Care. 2023;29(5):423–9. 10.1097/MCC.0000000000001082. 37641510 10.1097/MCC.0000000000001082

[28] Adrie C, Garrouste-Orgeas M, Ibn Essaied W, Schwebel C, Darmon M, Mourvillier B, et al. Attributable mortality of ICU-acquired bloodstream infections: impact of the source, causative micro-organism, resistance profile and antimicrobial therapy. J Infect. 2017;74(2):131–41. 10.1016/j.jinf.2016.11.001. 27838521 10.1016/j.jinf.2016.11.001

[29] Abe R, Oda S, Sadahiro T, Nakamura M, Hirayama Y, Tateishi Y, et al. Gram-negative bacteremia induces greater magnitude of inflammatory response than gram-positive bacteremia. Crit Care. 2010;14(2):R27. 10.1186/cc8898. 20202204 10.1186/cc8898 PMC2887127

[30] Nejtek T, Müller M, Moravec M, Průcha M, Zazula R. Bacteremia in patients with sepsis in the ICU: does it make a difference? Microorganisms. 2023;11(9):2357. 10.3390/microorganisms11092357. 37764201 10.3390/microorganisms11092357 PMC10534394

[31] Oliveira AM, Oliveira A, Vidal R, Gonçalves-Pereira J. Infectious foci, comorbidities and its influence on the outcomes of septic critically ill patients. Microorganisms. 2024;12(8):1705. 10.3390/microorganisms12081705. 39203547 10.3390/microorganisms12081705 PMC11357211

[32] Gupta E, Kumar S, Srivastava VK, Saxena J, Siddiqui AJ, Mehta S, et al. Unravelling the differential host immuno-inflammatory responses to *Staphylococcus aureus* and *Escherichia coli* infections in sepsis. Vaccines. 2022;10(10): 1648. 10.3390/vaccines10101648. 36298513 10.3390/vaccines10101648 PMC9610428

[33] Surbatovic M, Popovic N, Vojvodic D, Milosevic I, Acimovic G, Stojicic M, et al. Cytokine profile in severe Gram-positive and Gram-negative abdominal sepsis. Sci Rep. 2015;5:11355. 10.1038/srep11355. 26079127 10.1038/srep11355 PMC4468818

[34] Järvisalo MJ, Hellman T, Uusalo P. Mortality and associated risk factors in patients with blood culture positive sepsis and acute kidney injury requiring continuous renal replacement therapy—a retrospective study. PLoS ONE. 2021;16(4): e0249561. 33819306 10.1371/journal.pone.0249561 PMC8021149

[35] Xie P, Wang W, Dong M. Long-term mortality predictors of ICU fungaemia. Epidemiol Infect. 2021;149: e241. 10.1017/S0950268821002235. 34658330 10.1017/S0950268821002235 PMC8637461

[36] Palomar M, Álvarez-Lerma F, Riera A, Díaz MT, Torres F, Agra Y, Larizgoitia I, Goeschel CA, Pronovost PJ, Bacteremia Zero Working Group. Impact of a national multimodal intervention to prevent catheter-related bloodstream infection in the ICU: the Spanish experience. Crit Care Med. 2013;41(10):2364–72. 10.1097/CCM.0b013e3182923622. 23939352 10.1097/CCM.0b013e3182923622

[37] Osman MF, Askari R. Infection control in the intensive care unit. Surg Clin North Am. 2014;94(6):1175–94. 10.1016/j.suc.2014.08.011. 25440118 10.1016/j.suc.2014.08.011

[38] Chaves F, Garnacho-Montero J, Del Pozo JL, et al. Diagnosis and treatment of catheter-related bloodstream infection: clinical guidelines of the Spanish Society of Infectious Diseases and Clinical Microbiology and (SEIMC) and the Spanish Society of Spanish Society of Intensive and Critical Care Medicine and Coronary Units (SEMICYUC). Med Intensiva. 2018;42(1):5–36. 10.1016/j.medin.2017.09.012. 29406956 10.1016/j.medin.2017.09.012

